# Obesity/Weight Gain and Breast Cancer Risk: Findings From the Japan Collaborative Cohort Study for the Evaluation of Cancer Risk

**DOI:** 10.2188/jea.JE20120102

**Published:** 2013-03-05

**Authors:** Sadao Suzuki, Masayo Kojima, Shinkan Tokudome, Mitsuru Mori, Fumio Sakauchi, Kenji Wakai, Yoshihisa Fujino, Yingsong Lin, Shogo Kikuchi, Koji Tamakoshi, Akiko Tamakoshi

**Affiliations:** 1 Department of Public Health, Nagoya City University Graduate School of Medical Sciences, Nagoya, Japan; 1 名古屋市立大学大学院医学研究科 公衆衛生学分野; 2 National Institute of Health and Nutrition, Tokyo, Japan; 2 国立健康・栄養研究所; 3 Department of Public Health, Sapporo Medical University School of Medicine, Sapporo, Japan; 3 札幌医科大学医学部公衆衛生学; 4 Department of Preventive Medicine, Nagoya University Graduate School of Medicine, Nagoya, Japan; 4 名古屋大学大学院医学研究科予防医学; 5 Department of Preventive Medicine and Community Health, University of Occupational and Environmental Health, Kitakyushu, Fukuoka, Japan; 5 産業医科大学公衆衛生学; 6 Department of Public Health, Aichi Medical University School of Medicine, Nagakute, Aichi, Japan; 6 愛知医科大学公衆衛生学; 7 Department of Nursing, Nagoya University School of Health Sciences, Nagoya, Japan; 7 名古屋大学医学部保健衛生学科; 8 Department of Public Health, Hokkaido University Graduate School of Medicine, Sapporo, Japan; 8 北海道大学大学院医学研究科公衆衛生学

**Keywords:** breast cancer, obesity, weight gain, cohort study

## Abstract

**Background:**

We analyzed data from the Japan Collaborative Cohort Study (36 164 women aged 40–79 years at baseline in 1988–1990 with no previous diagnosis of breast cancer and available information on weight and height) to examine the association between baseline body mass index (BMI)/weight gain from age 20 years and breast cancer risk in a non-Western population.

**Methods:**

The participants were followed prospectively from enrollment until 1999–2003 (median follow-up: 12.3 years). During follow-up, breast cancer incidence was mainly confirmed through record linkage to population-based cancer registries. A Cox proportional hazards model was used to calculate hazard ratios (HRs) and 95% CIs for the association between breast cancer risk and body size.

**Results:**

In 397 644.1 person-years of follow-up, we identified 234 breast cancer cases. Among postmenopausal women, the adjusted HR increased with BMI, with a significant linear trend (*P* < 0.0001). Risk was significantly increased among women with a BMI of 24 or higher (HR: 1.50, 95% CI: 1.09–2.08 for BMI of 24–28.9, and 2.13, 1.09–4.16 for BMI ≥ 29) as compared with women with a BMI of 20 to 23.9. Weight gain after age 20 years and consequent overweight/obesity were combined risk factors for postmenopausal breast cancer risk. This combined effect was stronger among women aged 60 years or older. However, the HRs were not significant in premenopausal women.

**Conclusions:**

Our findings support the hypothesis that weight gain and consequent overweight/obesity are combined risk factors for breast cancer among postmenopausal women, particularly those aged 60 years or older.

## Abstract (ja)

**【背景】:**

日本共同コホート研究（JACC研究）の、1988－1990年をベースラインとした40－79歳の女性36164人（乳がんの既往がなく、身長や体重のデータが揃っているもの）のデータを用いて、肥満指数（BMI）と20歳からの体重増加が乳がんと関連しているかを、欧米人以外の集団で検討するために分析した。

**【方法】:**

対象者を1999－2003年まで追跡した（追跡期間の中央値は12.3年）。追跡期間中、乳がん罹患は主として地域がん登録と記録照合して確認した。コックスの比例ハザードモデルを用い、体格と乳がんの関連についてのハザード比と95％信頼区間を計算した。

**【結果】:**

397644.1人年の追跡で、234例の乳がん罹患があった。閉経後の女性では、BMIの上昇に伴い補正したハザード比は有意な線形傾向をもって上昇した（p<0.0001）。BMIが20から23.9の女性と比べて、24以上では有意にリスクは高かった（BMIが24-28.9ではハザード比：1.50、95％信頼区間：1.09－2.08、29以上ではハザード比：2.13、95％信頼区間：1.09－4.16）。20歳以降の体重増加とそれに伴う肥満・過体重が重なると、閉経後の乳がんのリスクとなった。このリスクの重なりは、60歳以上の女性でより強く観察された。閉経前の女性については、体格と乳がん発生の間に有意な結果は観察されなかった。

**【結論】:**

我々の得た知見は、体重増加とそれに伴う肥満・過体重が重なると、閉経後の、特に60歳以上の女性で乳がんのリスクになるという仮説を支持した。

## INTRODUCTION

Since the early 1990s, breast cancer has been the most frequently diagnosed cancer in Japanese women.^1^ Among women, the mortality rate of breast cancer is second only to that of stomach cancer. The recent continuous increase in breast cancer incidence has been an important public health concern in Japan, and the attention devoted to obesity/weight gain as a risk factor for breast cancer has also increased. Obesity is a well-known risk factor for postmenopausal breast cancer.^2^^–^^4^ Numerous epidemiologic studies have reported positive associations between obesity and breast cancer risk among white,^5^^–^^10^ African-American,^11^^–^^13^ and East Asian women.^14^^–^^17^ Furthermore, weight gain has been reported as an independent risk factor.^8^^,^^9^^,^^11^^,^^17^^–^^21^ Several studies have reported an inverse association between body weight in early adulthood and breast cancer incidence.^17^^,^^19^^,^^20^ However, the association has been somewhat inconsistent among premenopausal women. Obesity is associated with a decreased risk of breast cancer among white women,^4^^,^^10^^,^^22^^–^^24^ although accumulating evidence suggests that the inverse association is limited to women with estrogen receptor- and progesterone receptor-positive tumors.^25^^–^^28^ Studies of non-white racial/ethnic groups are more limited, and the results are mixed.

To assist in cancer prevention, we analyzed data from a large cohort study—the Japan Collaborative Cohort (JACC) Study—which included 64 327 Japanese women, to examine the association of baseline body mass index (BMI)/weight gain with breast cancer risk, considering menopausal status at baseline. We also investigated the interaction of age on this association.

## METHODS

### Study population

We analyzed data from the JACC Study, a prospective cohort study that evaluated cancer risk associated with lifestyle factors among the Japanese population. The study has been described in detail previously.^29^^,^^30^ In brief, the JACC Study was initiated in 1988–1990 and included 110 792 individuals (46 465 men and 64 327 women) aged 40 to 79 years from 45 areas throughout Japan. All participants were subsequently followed for all-cause mortality. In addition, study participants living in 24 areas with cancer registry systems were followed for cancer incidence.

Of the 64 327 women in the baseline cohort, 38 720 lived in the 24 areas where data on cancer incidence were available. The present study excluded 248 women who reported a previous diagnosis of breast cancer and 2308 women who did not provide information on height or weight at baseline. Thus, 36 164 women were included in the present analysis.

Informed consent was obtained from the participants in the form of signatures on the cover pages of the questionnaires, with the exception of those in a few study areas where informed consent was provided at the group level after the aims and data confidentiality had been explained to community leaders. The Ethics Board of Sapporo Medical University approved our study.

### Exposure assessment

As a relative indicator of body weight, BMI was calculated as weight in kilograms divided by the square of the height in meters (kg/m^2^). Information regarding weight and height was obtained from the self-reported questionnaire. Change in weight from age 20 years to the baseline measurement was calculated as the difference in the reported values at baseline among 20 418 women whose information on weight at age 20 years was available. We did not use BMI for age 20 years because we did not have access to height information at that age.

Information on other potential breast cancer risk factors such as family history of breast cancer, tobacco and alcohol use, age at menarche, marital status, parity, age at first birth, menopausal status, hormone use, and physical activity was collected in the baseline questionnaire. We have no information after baseline, including information on body size or menopausal status.

### Follow-up and identification of breast cancer cases

We followed the study participants from enrollment until 1999–2003. During this period, a population registry was used in each municipality to ascertain the residential status and vital status of the participants. In Japan, the Family Registration Law requires registration of all deaths, which theoretically provides complete mortality data. Breast cancer incidence was confirmed mainly through record linkage to population-based cancer registries in each area. To complete the incidence data, we also conducted a systematic review of death certificates and medical records at major local hospitals in some areas.

During the study period, 1799 (5.0%) participants were lost to follow-up due to moving out of their designated study areas. Among the 234 breast cancer cases, no information on diagnosis was available for 13 (5.6%), ie, they were identified with death certification only (DCO). The world standard for DCO in cancer registration is less than 10%. The mortality-to-incidence ratio for breast cancer was 0.262 (58/221) in the cohort covered by cancer registries, which was within the range calculated using available data from population-based cancer registries in Japan (0.20–0.30). We estimated that 36.5 cases of incident breast cancer were not included in the cancer registries.

### Statistical analysis

For each cohort subject, person-years of follow-up were counted as time from enrollment to diagnosis of breast cancer, death from any cause, or end of follow-up (1999–2003), whichever occurred first. For breast cancer cases ascertained only by death certificates, person-years of follow-up were calculated from enrollment to death from breast cancer. Those who died from causes other than breast cancer or who moved out of the study areas were treated as censored cases. We used a Cox proportional hazards model to estimate hazard ratios (HRs) and 95% CIs for the association of breast cancer risk with baseline BMI/weight change. Women were divided into 5 categories, using baseline BMI (in accordance with the World Health Organization classification)^31^: less than 18.5, 18.5–19.9, 20–23.9, 24–28.9, and 29 kg/m^2^ or higher. Furthermore, BMI was entered directly to evaluate the linear trend of relative weight. The effect of age on the association between BMI and breast cancer risk was examined by analyzing the relationship between age and BMI. Finally, to investigate the combined effect of baseline BMI and weight change from age 20 years, we recategorized the participants into 4 groups using the following cutoff points: baseline BMI less than 24 kg/m^2^ and weight gain of less than 10 kg from age 20 years to the baseline measurement.

We evaluated the association using age-adjusted and multivariable models with adjustment for age (using 10-year age groups), tobacco smoking (never, past, current, or unknown), alcohol consumption (never, past, current, or unknown), age at menarche (<15, 15–16, ≥17 years, or unknown), education level (attended school until age <16, 16–18, ≥19 years, or unknown), parity (nulliparous, 1, 2–3, ≥4 births, or unknown), age at first birth (<22, 22–23, 24–25, ≥26 years, or unknown), menopausal status (premenopausal at baseline, <45, 45–49, or ≥50 years), use of exogenous female hormone (yes, no, or unknown), first-degree family history of breast cancer (yes, no, or unknown), and physical activity categories^32^ (4 groups using the following cutoff points of physical activity: daily walking <1 h and exercise time <1 h a week, or unknown). All analyses were performed with regard to menopausal status and stratified by 6 study areas (Hokkaido and Tohoku, Kanto, Chubu, Kinki, Chugoku, and Kyushu).

We repeated the analysis after excluding the first 2 years of follow-up, during which 38 cases of breast cancer were diagnosed. All *P* values were 2-sided, and a *P* value less than 0.05 was considered to indicate statistical significance. All regression analyses were performed using the PROC PHREG procedure of SAS Version 9.1 (SAS Institute, Cary, NC, USA). Study areas were not incorporated in the Cox model with other potential confounders but were adjusted for using the strata option in the PHREG procedure.

## RESULTS

Average age and BMI (SD) at baseline of the 36 164 women were 57.8 (10.0) years and 22.9 (3.1) kg/m^2^, respectively. In 397 644.1 person-years of follow-up (median follow-up time, 12.3 years), we identified 234 breast cancer cases. Table 1 shows the distribution of risk factors for breast cancer in association with BMI. Women with a BMI less than 18.5 were older and more likely to be nulliparous and unmarried. The 2 extreme BMI groups had higher percentages of smokers and lower percentages of drinkers. Groups with higher BMI at baseline had increased weights at age 20 years and greater weight gain from age 20 years to baseline. However, the difference in weight at age 20 years between the 2 extreme BMI groups was relatively small (46.5 kg vs 52.2 kg), and weight change from age 20 years (−6.3 kg vs 17.1 kg) was a stronger contributor to body size at baseline. The average (SD) overall change in weight during the period was 2.7 (8.2) kg.

**Table 1.** Baseline characteristics associated with BMI in the JACC Study

Characteristics	BMI at baseline				

	<18.5	18.5–19.9	20–23.9	24–28.9	≥29
Number, *n* (row%)	2373 (6.6%)	3654 (10.1%)	18 231 (50.4%)	10 737 (29.7%)	1169 (3.2%)
Height (cm)	152.0 ± 7.0	151.0 ± 5.8	151.3 ± 5.5	150.7 ± 5.6	149.3 ± 6.4
BMI	17.4 ± 1.0	19.3 ± 0.4	22.0 ± 1.1	25.8 ± 1.3	31.0 ± 2.0
Weight at age 20 years (kg)	46.5 ± 6.1	47.8 ± 5.7	49.6 ± 6.2	51.0 ± 6.6	52.2 ± 6.8
Weight change^a^ (kg)	−6.3 ± 5.9	−3.7 ± 5.4	1.1 ± 6.3	7.8 ± 7.0	17.1 ± 8.3
Age at inclusion (years)	61.3 ± 10.8	58.5 ± 10.7	57.1 ± 10.0	57.9 ± 9.3	58.3 ± 9.3
Age at menarche (years)	15.2 ± 1.8	15.0 ± 1.8	14.9 ± 1.8	14.8 ± 1.8	14.9 ± 1.9
Age at first birth (years)	25.4 ± 3.5	25.2 ± 3.3	25.0 ± 3.2	24.9 ± 3.2	25.0 ± 3.5
Age at menopause (years)	48.2 ± 4.9	48.5 ± 4.5	48.8 ± 4.6	48.7 ± 4.8	48.5 ± 5.1
Years of education	16.5 ± 2.2	16.6 ± 2.1	16.7 ± 2.1	16.3 ± 2.0	16.0 ± 2.1
Nulliparous, *n* (%)	144 (6.6%)	175 (5.2%)	700 (4.1%)	404 (4.0%)	53 (4.9%)
Not married, *n* (%)	69 (3.4%)	61 (1.9%)	227 (1.4%)	111 (1.2%)	20 (2.0%)
Exogenous female hormone use, *n* (%)	124 (6.2%)	160 (5.2%)	792 (5.1%)	471 (5.2%)	61 (6.1%)
Family history of breast cancer, *n* (%)	30 (1.3%)	42 (1.2%)	269 (1.5%)	167 (1.6%)	13 (1.1%)
Current smoker, *n* (%)	162 (7.6%)	201 (6.2%)	779 (4.7%)	470 (4.8%)	81 (7.7%)
Current drinker, *n* (%)	453 (20.4%)	790 (23.1%)	4250 (24.8%)	2444 (24.2%)	223 (20.5%)

BMI, body mass index.

Mean (SD) or %, calculated from subjects with no missing data for any variable.

^a^Difference in body weight at age 20 years and baseline.

Table 2 shows breast cancer risk associated with baseline BMI in relation to menopausal status. After adjustment for potential confounding factors, neither a significant HR nor a linear trend was observed among the 8131 premenopausal women. In contrast, among 28 033 postmenopausal women, the adjusted HR increased with BMI and showed a significant linear trend (*P* < 0.0001). Furthermore, significantly increased risk was observed among women with a BMI of 24 or higher (HR: 1.50, 95% CI: 1.09–2.08 for BMI of 24–28.9; 2.13, 1.09–4.16 for BMI ≥29) as compared with those with a BMI of 20 to 23.9. The adjusted HRs per 5-kg/m^2^ increment in BMI among pre- and postmenopausal women were 0.95 (95% CI: 0.60–1.50) and 1.68 (95% CI: 1.34–2.01), respectively.

**Table 2.** Hazard ratios for breast cancer associated with BMI in the JACC Study

BMI	Cases	Person-years	Age-adjusted		Multivariate^a^	
	
			Hazard ratio	95% CI	Hazard ratio	95% CI
Premenopausal women						
<18.5	3	4799	0.89	(0.28–2.89)	0.82	(0.25–2.68)
18.5–19.9	6	10 327	0.83	(0.35–1.97)	0.78	(0.33–1.84)
20–23.9	39	55 363	1.00	Reference	1.00	Reference
24–28.9	13	25 975	0.71	(0.38–1.33)	0.76	(0.40–1.43)
≥29	1	2453	0.54	(0.07–3.97)	0.62	(0.08–4.58)
*P* for trend			0.97		0.82	
Postmenopausal women						
<18.5	7	19 412	0.71	(0.33–1.55)	0.64	(0.30–1.40)
18.5–19.9	7	28 831	0.47	(0.22–1.02)	0.46	(0.21–1.00)
20–23.9	77	146 684	1.00	Reference	1.00	Reference
24–28.9	71	93 372	1.47	(1.06–2.03)	1.50	(1.09–2.08)
≥29	10	10 427	2.00	(1.03–3.89)	2.13	(1.09–4.16)
*P* for trend			<0.0001		<0.0001	

BMI, body mass index.

^a^Adjusted for age, height, age at menarche, age at menopause (among postmenopausal women only), years of education, parity, marital status, use of exogenous female hormone, first-degree family history of breast cancer, smoking status, alcohol drinking, physical activity, and study area.

To observe the effect of age on the association between BMI and breast cancer risk among postmenopausal women, we calculated the HR for a 5-kg/m^2^ increment in BMI in younger (40–59 years) and older (60–79 years) age groups. The older group had a higher HR (2.00, 95% CI: 1.48–2.70) than the younger group (1.37, 95% CI: 0.96–1.96) for a 5-kg/m^2^ increment of BMI, after adjustment for potential confounders.

An effect of weight gain between age 20 years and baseline on breast cancer risk was observed only among postmenopausal women. The HR (95% CI) for 1 increment of weight gain was 1.04 (1.01–1.07). Among premenopausal women it was 0.99 (0.94–1.04) and not significant.

The combinatorial effect of baseline BMI and weight change between age 20 years and baseline was examined to evaluate the effect of these factors separately (Table 3). In premenopausal women, no significant HR or association was found. Conversely, in postmenopausal women, only those with a baseline BMI of 24 or higher and weight gain of at least 10 kg from age 20 years to baseline had a significant HR (2.55, 95% CI: 1.47–4.42), as compared with those with a baseline BMI of less than 24 and a weight gain of less than 10 kg from age 20 years to baseline. These findings indicate that weight gain after age 20 years and consequent overweight/obesity are combined risk factors for breast cancer among postmenopausal women. This combined effect was particularly strong in older women (HR: 4.08, 95% CI: 1.88–8.88). In addition, weight at age 20 years was not a significant predictor of breast cancer after adjustment for height at baseline and other potential confounders among premenopausal and postmenopausal women in this study. Furthermore, similar results were obtained after excluding the 33 breast cancer cases that occurred during the first 2 years of follow-up (data not shown).

**Table 3.** Multivariate hazard ratios for breast cancer associated with baseline BMI and weight change among postmenopausal women in the JACC Study

Weight change from age 20 years	Baseline BMI <24		Baseline BMI ≥24	
	
	Hazard ratio	95% CI	Hazard ratio	95% CI
Premenopausal women				
Loss, unchanged, or gain of <10 kg	1.00	Reference	0.94	(0.35–2.55)
Gain of ≥10 kg	0.53	(0.07–3.96)	1.88	(0.85–4.16)
Postmenopausal women				
Loss, unchanged, or gain of <10 kg	1.00	Reference	1.34	(0.69–2.58)
Gain of ≥10 kg	0.99	(0.24–4.19)	2.55	(1.47–4.42)

BMI, body mass index.

Adjusted for age, height, age at menarche, years of education, parity, marital status, use of exogenous female hormone, first-degree family history of breast cancer, smoking status, alcohol drinking, physical activity, and study area.

## DISCUSSION

To our knowledge, this is the first prospective report from Japan on the association between obesity/weight gain and breast cancer risk by age group. Our findings revealed a significant association between BMI/weight gain and postmenopausal breast cancer risk, particularly among older women. For postmenopausal women, especially those aged 60 years or older, weight gain after age 20 years and consequent overweight/obesity were identified as combined risk factors for breast cancer, after adjusting for potential confounders. In other words, being overweight or obese at baseline was a much greater risk factor among women who were postmenopausal, were aged 60 years or older, and had gained at least 10 kg from age 20 years to baseline.

Our results for postmenopausal women are consistent with those obtained in a number of studies worldwide. The adjusted HR per 5-kg/m^2^ increment in BMI in the present study (1.68) was slightly higher than the summary risk ratios from a meta-analysis^4^ of studies conducted in the Asia-Pacific (1.31), North America (1.15), and Europe and Australia (1.09). Breast cancer prevention via weight control is expected to be more effective among postmenopausal women in the Asia-Pacific region. With regard to cancer pathogenesis, the increased risk in overweight/obese postmenopausal women is due to the fact that adipose tissue is the major source of estrogenic hormones after menopause.^33^^,^^34^ Furthermore, our results conform with those of an earlier report showing that adult weight gain might be better than cross-sectional BMI as an adiposity index.^35^

In contrast, we did not observe any significant association between BMI/weight change and breast cancer risk among premenopausal women. In our cohort, age at baseline was 40 years or older; thus, follow-up did not completely cover the premenopausal period. A previous study reported an inverse association between BMI and breast cancer risk among white women. One hypothesis is that young overweight women are more likely to have anovulatory cycles with less cumulative exposure to endogenous estrogen.^36^^,^^37^ Another hypothesis is that there is greater clearance of estrogen by the liver in young overweight women.^38^ These hypotheses are strengthened by results from studies suggesting that the inverse associations are limited to women with tumors that are estrogen receptor- and progesterone receptor-positive.^25^^–^^28^ Thus, the heterogeneity of pathologic types among premenopausal breast cancer weakens the association and possibly explains the inconsistent results among non-white racial/ethnic groups. This heterogeneity of cancer etiology in relation to BMI and receptor type makes cancer prevention in premenopausal women difficult and of less practical importance. Further investigations of cancer pathogenesis are needed among non-white racial/ethnic groups.

A major advantage of the present study was its prospective design, which may avoid the possibility of recall bias inherent to case-control studies. Moreover, information on other breast cancer risk factors was included, and potential confounding factors were controlled in analyses of the association.

This study has some limitations that should be considered when interpreting our results. First, because we did not have updated information on menopausal status, which would modify the association between BMI/weight change and breast cancer, the possibility of misclassification of menopausal status at breast cancer onset should be considered. Such misclassification would be problematic in premenopausal women, since recently menopausal women would be misclassified as premenopausal during the follow-up period. Such misclassification could partly explain the inconsistent results from several studies of the association between body size and breast cancer among premenopausal women. Studies of younger women with updated information on menopausal status should be initiated among premenopausal women. However, this limitation is a minor concern for postmenopausal women. Changes during follow-up, especially those related to lifestyle, might alter the results. However, many risk factors, such as marriage status, number of children, and family history of breast cancer, would be unlikely to change after age 40. To our knowledge, substantial changes in risk factors for breast cancer related to BMI have not been reported.

Second, because we used simple questionnaires at baseline only, we have data at only 2 time points, ie, age 20 years and baseline. We did not have data on the time period of weight gain, which would provide useful information for recommendations. Lack of information on weight gain around menopause would also weaken the association among premenopausal women. Furthermore, weight at age 20 years is retrospective information and may be systematically biased among women at extremes of body size. However, these data were obtained before breast cancer diagnosis, and therefore any misclassification is not likely to be differential.

The accuracy of cancer identification in the present study was not ideal. We estimated that 36.5 cases of incident breast cancer were not included in our follow-up, and this number is not inconsiderable. However, these cases would be independent of body size; thus, estimated HRs would tend toward the null.

In summary, our findings support the hypothesis that a weight gain of 10 kg or more and consequent overweight/obesity (BMI ≥24) are combined risk factors for breast cancer among Japanese postmenopausal women, particularly those aged 60 years or older. Thus, to prevent breast cancer, weight gain after age 20 years should be avoided and weight control should be increasingly emphasized with increasing age. The association between body size and premenopausal breast cancer was not clear in the present study and varies across studies; thus, optimal weight for breast cancer prevention cannot be specified at this time.

## ONLINE ONLY MATERIALS

Abstract in Japanese.
