# Supplementary material for: Obesity/Weight Gain and Breast Cancer Risk: Findings From the Japan Collaborative Cohort Study for the Evaluation of Cancer Risk
Source: J Epidemiol. 2013 Mar 5;23(2):139–45. doi: 10.2188/jea.JE20120102 (PMC3700246; doi:10.2188/jea.JE20120102)
Supplement: Abstract in Japanese. [file je-23-139-s001.pdf]

肥満／体重増加と乳がんリスク：がんリスクの評価のためのJACC研究からの知見

鈴木貞夫[1]、小嶋雅代[1]、徳留信寛[2]、森満[3]、坂内文男[3]、若井建志[4]、藤野善久[5]、林櫻松[6]、菊地正悟[6]、玉腰浩司[7]、玉腰暁子[8]、JACC研究グループ

[1]名古屋市立大学大学院医学研究科　公衆衛生学分野、[2]国立健康・栄養研究所、[3]札幌医科大学医学部公衆衛生学、[4]名古屋大学大学院医学研究科予防医学、[5]産業医科大学公衆衛生学、[6]愛知医科大学公衆衛生学、[7]名古屋大学医学部保健衛生学科、[8]北海道大学大学院医学研究科公衆衛生学

【背景】 日本共同コホート研究（JACC研究）の、1988－1990年をベースラインとした40－79歳の女性36164人（乳がんの既往がなく、身長や体重のデータが揃っているもの）のデータを用いて、肥満指数（BMI）と20歳からの体重増加が乳がんと関連しているかを、欧米人以外の集団で検討するために分析した。

【方法】 対象者を1999－2003年まで追跡した（追跡期間の中央値は12.3年）。追跡期間中、乳がん罹患は主として地域がん登録と記録照合して確認した。コックスの比例ハザードモデルを用い、体格と乳がんの関連についてのハザード比と95％信頼区間を計算した。

【結果】 397644.1人年の追跡で、234例の乳がん罹患があった。閉経後の女性では、BMIの上昇に伴い補正したハザード比は有意な線形傾向をもって上昇した（p<0.0001）。BMIが20から23.9の女性と比べて、24以上では有意にリスクは高かった（BMIが24−28.9ではハザード比：1.50、95％信頼区間：1.09－2.08、29以上ではハザード比：2.13、95％信頼区間：1.09－4.16）。20歳以降の体重増加とそれに伴う肥満・過体重が重なると、閉経後の乳がんのリスクとなった。このリスクの重なりは、60歳以上の女性でより強く観察された。閉経前の女性については、体格と乳がん発生の間に有意な結果は観察されなかった。

【結論】 我々の得た知見は、体重増加とそれに伴う肥満・過体重が重なると、閉経後の、特に60歳以上の女性で乳がんのリスクになるという仮説を支持した。

キーワード：　乳がん、肥満、体重増加、コホート研究
